# Extraction and Characterization of Highly Gelling Low Methoxy Pectin from Cashew Apple Pomace

**DOI:** 10.3390/foods3010001

**Published:** 2013-12-23

**Authors:** Beda M. Yapo, Kouassi L. Koffi

**Affiliations:** 1 Subunit of Pedagogy in Biochemistry and Microbiology, Unit of Training and Research in Agroforestry, University of Jean Lorougnon Guédé, BP 150 Daloa, Cote d’Ivoire; 2 Polytechnique National Institute of Felix Houphouet Boigny, BP V79 Abidjan, Cote d’Ivoire; E-Mail: koffileonard@yahoo.fr

**Keywords:** cashew apple, low methoxy pectin, purification, molecular features, gelation

## Abstract

Investigation on the pectic substances of cashew (*Anacardium occidentale* L.) apple under different acid-extraction conditions (pH 1.0, 1.5, and 2.0) showed that more than 10%–25% of *A. occidentale* pectins (AOP) could be extracted, depending on the extractant strength. The extracted AOP contained high amounts of galacturonic acid (GalA: 69.9%–84.5%) with some neutral sugars of which rhamnose (Rha: 1.3%–2.5%), arabinose (Ara: 2.6%–5.4%), and galactose (Gal: 4.7%–8.6%) were the main constituents. The degree of methoxylation (DM) was in the range of 28%–46% and was only slightly affected by the extractant strength, thereby indicating isolation of naturally low methoxy pectins (LMP). In terms of gelling capability, AOP yielded firmer Ca^2+^-mediated LMP gels than commercial citrus LMP with comparable DM. Cashew apple pomace, therefore, appears to be a potentially viable source for possible production of “non-chemically or enzymatically-tailored” LMP.

## 1. Introduction

Pectic substances are a family of at least eight polysaccharide types, present in the cell wall of all (if not most) higher plants, with complex macromolecular structures that would be made of no less than 17 monosaccharide types [1]. Pectins are generally viewed as gelling polysaccharides, from miscellaneous plant byproducts, which are mainly composed of α-d-galactopyranosyluronic acid (α-d-Gal*p*A) residues, partially methyl-esterified at *C*-6 position, and some neutral sugars, three of which (α-l-rhamnopyranose (α-l-Rha*p*), α-l-arabinofuranose, and β-d-galactopyranose) are typical monosaccharide residues. Various structural studies, with highly purified enzyme preparations, revealed that the glycosyl residues were not randomly distributed in the pectin macromolecules, but they were rather concentrated in different regions, which gave birth to two main building block copolymers, namely homogalacturonan (HG) and rhamnogalacturonan-I (RG-I). HG is an unbranched 1,4-α-d-Gal*p*A polymer which is partially methyl-esterified at *C*-6 position and sometimes acetyl-esterified at *O*-2 and/or *O*-3 positions. RG-I is a [→4)-α-d-Gal*p*A-(1→2)-α-l-Rha*p*-(1→]*n* polymer partly branched with diverse neutral sugar side chains. Common side chains of RG-I are 1,5-α-l-arabinan, 1,4-β-d-galactan, and arabinogalactan-I. They may, however, be ramified with more complex polysaccharide moieties, such as arabinogalactan-II and the rather scarce galactoarabinan [2]. 

To date, commercial pectins are only produced from citrus (lime, lemon, orange, and grapefruit) peels and apple pomace, two raw materials from the juice industry available in large quantities in western countries [3,4]. Their selection for this purpose is accounted for by the fact that dry citrus peel (15%–30%) and apple pomace (10%–15%) are pectin-rich sources. Moreover, acid-extracted citrus and apple pectins are usually characterized by high viscosity-average molecular weights (*M_v_*: 100–300 kDa), and high GalA (≥65%) and methoxy (8%–12%) contents. These features enable one to prepare good sugar-acid-mediated high methoxy pectin (HMP) gels in the presence of high sugar (especially sucrose) content (55%–65% wt). To manufacture low calorie gelling products, pectins with low methoxy content (<7%), which form Ca^2+^-induced gels whether sugar is added or not, are required. This compels the producers of commercial pectins to add to their pectin production processes a step of deesterification of the initially extracted HMP from citrus and apple byproducts. Commercial LMP, with degrees of methoxylation (DM) ≤ 50, are usually produced from HMP (DM > 50) using one of the following methods: chemical (base or acid) treatment at cold temperature (LMP with randomly distributed deesterified GalA units), ammonia treatment (amidated LMP with randomly distributed deesterified GalA units), fungal pectin methylesterase treatment (LMP with non-blockwise distribution of deesterified GalA units), and/or plant pectin methylesterase treatment (LMP with blockwise distribution of deesterified GalA units). This further operation brings about additional cost of production of LMP, especially when enzymatic procedures using highly purified pectin methylesterases are needed for this purpose. Thus, the import of commercial pectins, and particularly LMP, in emerging and developing countries, such as Côte d’Ivoire and neighboring regions (in Africa), to satisfy their demands represents an expensive enterprise with low added values to domestically manufactured pectin-containing gelling products and confections. Therefore, ready-to-eat jams, marmalades, preserves, and various other pectin-containing confections from the western food industry are by far widely preferred to home-made ones by local food firms. As a consequence, most of the few domestic gelling food plants went bankrupt, thereby increasing the jobless mass. To partially solve this serious problem, “new pectin sources” from diverse agrowastes are being searched for.

Cashew plant (*A. occidentale*, Anacardiaceae) is mainly cultivated for its fruit nut, commonly known as “cashew nut”, which almond is used as foodstuffs and cosmetic and pharmaceutical ingredients. From an annual production of 6000 tons in 1990 to 300,000–350,000 tons over the 2008/2012 period [5,6], Côte d’Ivoire has made a quantum leap, by becoming the second top producer of cashew nuts in the world, closely behind India, which has produced about 400,000 tons. Thus, cashew nut has become the second most important source of incomes, after cotton, for the Ivorian peasant farmers living in the savannah areas. The fruit is composed of two parts: the juicy peduncle (or cashew apple) and the cashew nut. Once the valuable nut has been separated from the apple, the latter, which accounts for 60%–90% of the fruit fresh weight, is usually not consumed, because of its astringent taste [7], caused by the presence of anacardic acid. The unutilized apple is generally left in plantations to rot, thus posing a serious problem of plant disease inoculum. Therefore, it is necessary to find a way of adding value to it in order to increase returns to farmers and circumvent the ecological problem it causes. We here reported on the purification of “non-chemically and enzymatically-tailored” LMP with high gelling capabilities from cashew apple pomace.

## 2. Experimental Section

### 2.1. Alcohol Insoluble Material Preparation

Fresh cashew apple fruits were donated by factory-made producers and sellers of cashew nuts (CAJOUCI, Korhogo, Côte d’Ivoire). Fruits were minced in a Kenwood mincer and immediately soaked in 3 volumes of boiling 80% (v/v) ethanol for 25 min and cooled to room temperature. Alcohol-insoluble material (AIM) was continuously washed with 70% (v/v) ethanol to remove free sugars, pigments, and other impurities as much as possible. The residue was then dried by solvent exchange (95% ethanol and acetone), placed in a fume hood for 5 h for the evaporation of residual acetone and oven-dried for 15–16 h. Dried AIM was ground in a hammer mill (Model 912, Winona Attrition Mill Co., Winona, MN, USA) to pass through a 12 mm-sized sieve and was kept under moisture-free conditions until use.

### 2.2. Pectin Production

Pectins were extracted from AIM by water acidified with 1 N HNO_3_ to three different extractant strengths (pH 1.0, 1.5, and 2.0), while the other extraction parameters, namely, solid to liquid extractant ratio, temperature, and time, were invariably kept to 1:25 (w/v), 75 °C, and 90 min, respectively. Two successive extractions were performed before discarding any remaining insoluble cell wall residue. At the end of each extraction, the slurry was clarified and the pectin extract was rapidly brought to pH 4 for stability. The first and second extracts were pooled and treated with 0.5 M imidazole buffer (pH 7) and extensively dialyzed against distilled water in 12,000 molecular weight cut-off tubing to readily and completely remove Ca^2+^-imidazole complexes [8]. The retentate was then concentrated to the desired solution quantity and precipitated in 3 volumes of 95% ethanol at 5 °C for 2 h. Pectin precipitates were washed two-times with 70% ethanol, followed by 95% ethanol and acetone, and kept for a while under a fume extractor (for residual acetone evaporation), and finally oven-dried at 40–45 °C for 15–16 h and weighed. The extraction of pectins was carried out in three independent runs for each selected pH value. Dried pectin flakes were finely ground to pass through 60-mesh (# 0.25 mm) size sifters and the *A. occidentale* pectin (AOP) flours obtained were canned in plastic containers and stored at room temperature under airless and moisture-free conditions pending analysis.

A standardized commercial citrus low methoxy pectin (CCLMP), Genupectin LM12CG (DM = 34%) (Hercules, Copenhagen, Denmark), and its purified (sucrose-free) form (PLM12CG) in our laboratory [9] were used for comparison purposes.

### 2.3. Characterization of Pectins

The pectin samples were first treated with a mixture of 1% (v/v) HCl/60% (v/v) ethanol (three times), and insolubles were exhaustively washed with 60% (v/v) ethanol to totally remove free sugars. This treatment indeed aimed at simultaneously removing free sugars and salts and converting all the carboxyl groups of pectin macromolecules to the free acid (-COOH) form, prior to correctly titrating them by 1 N NaOH solution. Pectins were analyzed for their glycosyl residue composition, esterification degree, molecular weight, and gel-forming capability. 

#### 2.3.1. Proximate Analyses

The protein content of the pectin extracts was colorimetrically determined at 750 nm by a Folin-phenol reagent assay [10] using bovin serum albumin standard. Calcium element was analyzed as previously reported [9], by flame atomic absorption spectrometry at 422.7 nm using an Aanalyst 300 spectrophotometer (Perkin-Elmer Corp., Norwalk, CT, USA).

#### 2.3.2. Analyses of the Glycosyl Residue Composition

To quantify the monosaccharide constituents of the different samples, AIM was hydrolyzed with 1 mol·L^−1^ H_2_SO_4_ (100 °C, 3 h) after pretreatment with 12 mol·L^−1^ H_2_SO_4_ (23 °C, 1 h) and purified pectins were directly hydrolyzed with 1 mol·L^−1^ H_2_SO_4_ (100 °C, 3 h) as previously reported [11]. 

The GalA content of AIM and purified pectins was colorimetrically quantified at 525 nm by a modified sulfamate-meta-hydroxydiphenyl assay using monoGalA standard [12].

The neutral sugars, which were liberated from the purified pectins, especially Gal, Ara, and Rha [13] were spectrophotometrically quantified at 340 nm using Megazyme assay kits (Megazyme International Ireland Ldt., Bray, Co. Wicklow, Ireland). The neutral sugars assays were based on the quantitative oxidation of Gal/Ara and Rha to corresponding lactonic derivatives (d-galactono-(1,4)-lactone for α-l-Ara and β-d-Gal and l-rhamno-(1,4)-lactone for α-l-Rha) in the presence of corresponding dehydrogenases (β-Gal dehydrogenase plus Gal mutarotase for α-l-Ara and β-d-Gal, and l-Rha dehydrogenase for α-l-Rha) and the coenzyme NAD^+^, which is stoichiometrically reduced to NADH with absorbance maximum at 340 nm. d-Gal was quantitatively differentiated from l-Ara by reading absorbances at different reaction times, namely after 6 min- and 12 min-reaction at room temperature, respectively. l-Rha was quantitatively determined after 1 h-reaction at room temperature. 

The relative proportion of RG-I to HG block copolymers was roughly estimated by calculating the molar ratio of Rha to GalA and the degree of branching (DBr) of the pectins rhamnosyl residues, with neutral sugars side chains, was estimated by Equation (1) [9]:

DBr (%) = 100 × Rha (mol%)/[Ara (mol%) + Gal (mol%)]
(1)

It is indicative, in pectin polymer, of the minimum number of Rha residues branched with Ara and Gal residues, irrespective of the length of Ara and/or Gal residues-containing side chains. Thus, the greater the molar quantity of (Ara + Gal) than Rha, the lower the value of DBr is and the higher the level of branching of the (RG-I domain of) pectin polymer.

The overall esterification degree of pectic samples was potentiometrically determined as previously described [14]. The acetylesterification degree (DAc) was colorimetrically measured at 510 nm by the hydroxamic acid assay using glucose (Glc) pentaacetate standard [15] and the DM was calculated by difference. All the measurements were performed in triplicates.

#### 2.3.3. Analysis of the Macromolecular Characteristics of Pectins

The intrinsic viscosities of the samples were determined as described previously [9]. Briefly, the viscosities (*η*) of the samples prepared at different solution concentrations were first estimated by means of capillary viscometric experiments using an Ubbelohde capillary viscometer (capillary No.: I; id: 0.63 mm; Schott-Geräte GmbH, Mainz, Germany) with a constant *K* of 0.01, immersed in a water bath thermostated at 25 °C. The solutions of the samples were prepared at seven different concentrations (*C*) (0.01, 0.03, 0.05, 0.08, 0.10, 0.15, and 0.20 g/100 mL) in aqueous solvent containing 90 mM sodium chloride, 10 mM sodium fluoride, and 1 mM Na_2_EDTA at pH 6.5. The sample solutions and solvent were filtered using 0.45 µm membrane filters (Millipore Corp., Bedford, MA, USA) before measurements. The solutions (15 mL) were pipetted into the capillary viscometer. Flow times were recorded with a stopwatch with a precision of ±0.1 s. The densities of the sample solutions were measured by a 25 mL-Gay-Lussac pycnometer (Boeco, Boeckel & Co. (GmbH & Co.), Hambourg, Germany). The viscosities of the sample solution (*η*) and solvent (*η*_s_) were calculated from the measured flow times and densities of solutions, followed by calculations of the specific viscosities (*η*_sp_) using Equation (2):

*η*_sp_ = (*η* − *η*_s_)/*η*_s_ (2)

The intrinsic viscosities ([*η*]) of the samples were finally estimated by plotting the reduced viscosities (*η*_sp_/*C*) *versus* concentration (*C*) and extrapolating to zero polysaccharide concentration (Equation (3)):

[*η*] = lim *η_sp_*/*C* *C* → 0 (3)

For each sample analyzed, experiments were carried out five times and the average values were taken for plotting.

The molecular weight of the pectins was analyzed by gel-filtration chromatography on a high resolution Superdex-200 HR 10/30 column (Amersham Biosciences Corp., NJ, USA). The same solvent specified above (90 mM sodium chloride + 10 mM sodium fluoride + 1 mM Na_2_EDTA at pH 6.5) was used as eluent and the polysaccharide concentrations in the eluate were monitored using a differential refractometer or refractive index detector (Waters Corp., Milford, MA, USA). A molecular weight kit of pullulan standards (*M_w_* ~ 6.0, 10.0, 21.7, 48.8, 113.0, 210.0, 393.0, and 805.0 kDa; *M_w_* / *M_n_* ~ 1.0–1.2; American Polymer Standards Corp., Mentor, OH, USA) and purified homogenous HG standards (*M_w_* ~ 60 and 100 kDa, *M_w_* / *M_n_* ~ 1.0–1.2) [16], with known intrinsic viscosity ([*η*]) and *M_w_* values, were used for calibration. To better estimate the *M_v_* of the pectins, the so-called universal calibration technique was used by plotting log ([*η*] × *M_w_*) *versus* the elution volume of standards. Analyses were done in triplicates.

### 2.4. Gelling Properties

The gelling capability of pectins was evaluated according to the “adapted SAG-method” of Food Chemical Codex (FCC) to LMP as fully described previously [9]. The final composition of gels was 1.0% pectin material for AOP (and PLM12CG and 1.4% for LM12CG in order to have similar GalA contents) and 30% sucrose at pH 3.0 and 23.8–31.6 mg Ca^2+^/g pectin. The calcium effect was excluded by using the stoichiometric ratio of binding (*R* = 2 [Ca^2+^]/[-COO^−^]), which described the relationship between the molar concentrations of Ca^2+^ ions and ionisable carboxyl groups of polygalacturonate on the basis of the pectin de-methylesterification degree (100 − DM) [14]. Pectin dispersions (1.0%–1.4%), containing calcium ions which concentration was varied according to the *R* value and 30% sucrose were prepared as follows: 

Briefly, mixtures of weighed amounts of pectin powder and a half amount of sucrose were dissolved in 100 mM sodium chloride solution, under gentle stirring, at room temperature for 15–16 h. The pH of the solutions obtained was fined-tuned to 3.0 using few drops of 0.25 M citric acid or sodium citrate buffer if necessary. The mixtures were then heated to boiling point (85 ± 2 °C) under stirring and an appropriate amount of a pre-heated calcium chloride dihydrate solution, prepared in 100 mM sodium chloride, was slowly added under vigorous stirring until the desired calcium content was reached. To prevent pre-gelation, when adding the calcium ion solution to mixtures containing sucrose, the other half amount of sucrose was dissolved in the amount of CaCl_2_·2H_2_O solution to be added. The pH of the mixtures was controlled and kept constant during gelation. The prepared gels were molded and allowed to cool to room temperature, and were finally rested for 24 h at 4 °C before measuring the strength (or firmness) with the help of a Ridgelimeter (Bulmer Food Co., UK). A thin layer of low viscosity paraffin oil was used to cover the exposed surface of the gels in order to minimize weight loss by water evaporation. Experiments were carried out three times for each pectin sample analyzed.

### 2.5. Statistical Analysis

All the data were statistically appraised by a single-factor analysis of variance (ANOVA), followed by the Bonferroni’s posthoc test for multiple comparisons, whenever applicable, using a GraphPad Prism V.3 software (GraphPad software Inc., San Diego, CA, USA). Means of different treatments were considered to be significantly different at *p* value < 0.05.

## 3. Results and Discussion

### 3.1. The Yield of Pectins Extracted from Cashew Apple Pomace

The alcohol insoluble material (AIM), which represented 12.9% ± 1.4% (for three independent measurements) of the weight of fresh cashew apple, was found to contain 35.6% ± 2.2% (anhydro-)GalA. This suggested that cashew apple pomace was a highly-rich pectin source, upon which studies needed to be conducted for possible production of commercially marketable pectin products. The yield of AOP extracted under different acid strengths ranged from 10.7% to 25.3% (Table 1). The pectin yield was significantly influenced by the extractant strength (*p* < 0.05), the lowest yield being obtained under more severe conditions (at pH 1.0). Nevertheless, the pectin yield was >10% whatever the extraction conditions used, suggesting that cashew apple pomace was a potentially viable source of marketable pectins. The overall high pectin yield observed (>10%), in different acid conditions, suggested that most pectic substances might originally be loosely bound within the cell wall. The yield of pectin obtained at pH 1.5 was comparable enough to the one (38.7%) reported by extraction of pectins from cashew apple with sodium hexametaphosphate (SHMP), followed by precipitation with acidified acetone [7]. It should be underlined that the SHMP-extracted cashew apple pectin has been found to contain high amounts of impurities, mainly from SHMP. 

Furthermore, some difficulties were encountered by trying to directly isolate the pectin products from the clarified extracts by alcohol-precipitation as usually processed, because this resulted in gum-like pectin coagulates, with high moisture content, which could hardly be oven-dried. This problem was also reported when high amounts of SHMP-extracted pectins from cashew apple were recovered by precipitation with acidified acetone [7]. We found that mineral salts, especially Ca^2+^, were abundant in the pectin polymers solubilized from the cashew apple pomace and were mainly responsible for the gummy appearance of pectin coagulates. Therefore, the clarified extracts were first treated with 0.5 M imidazole buffer for the formation of Ca^2+^-imidazole complexes, which were readily removed from the pectin extracts by extensive dialysis as suggested elsewhere [8], before precipitating pectin polymers with alcohol. This treatment was effective for producing pectin coagulates with low moisture content and hence readily oven-dried.

**Table 1:** Glycosyl residue composition, macromolecular features, and gelling capability of acid-extracted pectins from cashew apple pomace.

	AOP			CCLMP^d^	
	pH 1.0	pH 1.5	pH 2.0	LM12CG	PLM12CG
Yield (% DRM)	10.7 ± 0.9 a	25.3 ± 4.4 b	16.4 ± 2.1 c	ND	ND
GalA (% w/w)	69.9 ± 2.8 a	84.5 ± 4.2 b	75.2 ± 1.6 c	53.9 ± 1.1	75.8 ± 1.5
Rha (% w/w)	1.3 ± 0.1 a	1.9 ± 0.3 ab	2.5 ± 0.2 b	0.7 ± 0.1	1.1 ± 0.1
Ara (% w/w)	2.6 ± 0.2 a	3.8 ± 0.4 ab	5.4 ± 0.9 b	0.4 ± 0.1	0.4 ± 0.1
Gal (% w/w)	4.7 ± 0.3 a	5.5 ± 0.3 ab	8.6 ± 1.1 b	1.9 ± 0.2	3.9 ± 0.2
Glc (% w/w)	ND	ND	ND	0.5 ± 0.1	0.7 ± 0.1
Rha/GalA	2.2/100 a	2.7/100 ab	4.0/100 b	1.6/100	1.7/100
DBr (%)	18.2 ± 0.9	20.6 ± 2.5	18.1 ± 1.4	27.6	32.3
DM	28 ± 4 a	41 ± 7 ab	46 ± 3 b	34 ± 1	31 ± 1
DAc	Trace	3 ± 1	5 ± 1	Trace	Trace
Protein (% w/w)	2.8 ± 0.1 a	4.1 ± 0.5 ab	5.3 ± 0.2 b	0.9 ± 0.1	ND
Calcium (μmol/g)	114.7 ± 3.1 a	55.6 ± 1.9 b	79.8 ± 2.4 c	32.2 ± 0.9	20.1 ± 1.1
[*η*] (mL/g)	287 ± 4 a	398 ± 7 b	425 ± 5 c	ND	ND
*M_v_* (kDa)	76 ± 7 a	142 ± 11 b	117 ± 8 c	ND	ND
Gel strength (°FCC)	97 ± 5 a	128 ± 1 b	109 ± 3 b	105	104

Data are expressed as mean ± SD (*n* = 3). Mean values in the same line with different letters are significantly different (*p* < 0.05). AOP: *A. occidentale* pectin; CCLMP^d^: commercial citrus low methoxy pectin, data are from [9]; DRM: Dried raw material; ND: Not determined.

### 3.2. Chemical Features of Extracted Pectins

The glycosyl residue composition of the purified pectins is shown in Table 1. The GalA content of AOP ranged from 69.9% to 84.5%. In all cases, the GalA content was >65%, one of the quality features that should be fulfilled for marketing possibility. This showed that AOP were indeed GalA-rich pectins, and therefore were of a rather high purity. These quantities of GalA were significantly different from one another (*p* < 0.05), indicating that the amount of the basic constituent of pectins was affected by the extraction conditions. The decrease in the amount of GalA from 84.5% to 69.9%, as the extractant strength was increased from pH 1.5 to pH 1.0, suggested that either substantial degradation of solubilized GalA-rich HG block copolymers or extraction of greater quantities of NS-containing RG-I block copolymers (and probably other GalA-free sugar materials) from the pomace.

Furthermore, the remarkably high GalA content (85.0%) of the AOP isolated at pH 1.5, strengthened the idea that most pectic substances might originally be loosely bound within the cell wall of cashew apple, probably via Ca^2+^ cross-bridges between adjacent galacturonate sequences within the pectin polymers [8] and, therefore, they were readily extractable, not only by Ca^2+^-complexants such as CDTA, EDTA, SHMP to name a few, but also by extracting agents of higher strengths, especially diluted solutions of strong minerals acids such as HNO_3_, HCl, and H_2_SO_4_ [17]. 

The three typical neutral sugars of pectic substances, namely, Rha: 1.3%–2.5%, Ara: 2.6%–5.4%, and Gal: 4.7%–8.6% were detected, in all the three AOP extracts, in moderately different amounts from one sample to another. This substantiated that the differences observed above, in the amount of the basic constituent (GalA) of pectin polymers, were not likely to be due to the presence of much more neutral sugar-containing RG-I block copolymers, but very probably to notable degradation of GalA-rich copolymers under more severe extraction (acid) conditions. Galactose was the major monosaccharide present, suggesting that the RG-I regions of AOP were mainly branched with galactan and/or arabinogalactan side chains. 

The relative proportions of HG to RG-I block copolymers, as judged by the molar ratio of Rha to GalA, suggested that the pectin HG building blocks were dominant over RG-I blocks in all the purified AOP and CCLMP. The degree of branching of the three AOP was not significantly influenced by the extraction conditions. However, AOP generally appears to be slightly more branched with neutral sugars than CCLMP.

Protein fraction and elemental calcium were also detected in all the three pectin isolates. The protein and calcium contents were in the range of 2.8%–5.3% and 55.6–114.7 μmol/g, respectively. Their amounts seemed to be moderately affected by the extraction conditions.

### 3.3. Degree of Esterification

The DM of AOP was in the range of 28–46 (Table 1), indicating extraction of LMP. The pectin DM was also affected by the extraction conditions. This showed that isolation of LMP did not result from acid-deesterification of the pectic substances in the cell wall of cashew apple pomace. It seemed that these pectin polymers were originally weakly methyl-esterified within the cell wall and probably abundantly present in the middle lamellae through Ca^2+^ cross-bridges between adjacent unesterified galacturonate sequences of the pectin HG block copolymers. LMP have also been extracted from other pectin sources, such as olive fruit pomace [18], sunflower head residues [19], and yellow passion fruit rind [9,20]. It is generally believed that natural LMP macromolecules within the cell wall result from nascent HMP following the activity of pectin-methyl esterases. The DAc was rather low in all the extracted pectins (<5%). 

### 3.4. Macromolecular Features

The [*η*] and *M_v_* of AOP varied from 287 to 425 mL/g and from 76 to 142 kDa, respectively (Table 1). The two macromolecuclar parameters were significantly influenced by the extraction conditions (*p* < 0.05). The AOP isolated at pH 1.5 had the highest *M_v_* (142 kDa). The relatively low *M_v_* (76 kDa) of the AOP obtained at pH 1.0, compared with the other two pectin isolates obtained under milder acid conditions, could mainly be explained by higher degradation, principally via the neutral sugar-containing RG-I regions, of solubilized pectin polymers under more severe acid condition as suspected from the glycosyl residue composition. It has indeed been shown that the neutral sugar-side chains of pectin macromolecules are the major contributors to the molecular weight [21]. Moreover, the rather low [*η*] of the pH 1.0-AOP suggested that this pectin polymer might have an overall extended conformation as dominantly imposed by its rigid rod-like HG regions, whereas the other two pectins, especially the pH 2.0-AOP, seemed to have random coil-like conformation, as judged by their substantially high [*η*] [11]. On the other hand, the pH 2.0-AOP, with higher [*η*] (425 mg/mL), exhibited lower *M_v_* than the pH 1.5-AOP. This might tentatively be explained by the presence, in the pH 2.0-AOP, of relatively higher amount of other polymers (e.g., proteins) which notably affected the [*η*], but not the *M_v_*, of the isolated pectin polymers.

### 3.5. Gelling Capability

The results of the gelling assays of AOP are summarized in Table 1. All the three AOP extracts were able to form Ca^2+^-mediated LMP gels. However, the gelling grade (97–128) was significantly affected by the extractions conditions (*p* < 0.05). The pH 1.5-AOP possessed the highest gelling capability, which could be ascribed to its considerably high GalA content (85%), with a greater number of unesterified GalA (galacturonate) block sequences, and *M_v_* (142 kDa); two intrinsic factors known to substantially impact on Ca^2+^-induced gelation of LMP [4,14]. These results showed that LMP extracted from cashew apple pomace, under specified conditions, can fulfill the required gelling grade (>100) for marketing possibility. Moreover, AOP was likely to form firmer gels than did CCLMP with similar DM. In terms of structural characteristics and gelling ability, the pH 1.0-AOP was comparable to CCLMP.

## 4. Conclusions

With a galacturonic acid content as high as 35% on a dry weight basis, cashew apple pomace was found to be a pectin-rich industrial byproduct. More than 10%–25% of highly gelling “non-chemically or enzymatically-tailored” low methoxy pectins, containing high amounts of galacturonic acid (70%–85%), can be produced from this raw material under optimized conditions. Therefore, the cashew apple pomace appears to be a commercially viable raw material for the possible production of low methoxy pectins required for manufacturing low calorie gelling food products.

## Abbreviations

AIM: alcohol-insoluble material
AOP: *Anacardium occidentale* pectins
CCLMP: commercial citrus low methoxy pectin
DAc: degree of acetylesterification (or acetylation)
DBr: degree of branching of pectin rhamnosyl units with neutral sugars
DM: degree of methoxylation (or methylesterification or methylation)
DRM: dried raw material
HG: homogalacturonan
HMP: high methoxy pectin or pectin with high methoxy content
LMP: low methoxy pectin or pectin with low methoxy content
RG-I: rhamnogalacturonan-I
SHMP: sodium hexametaphosphate
